# Understanding Cysteine Chemistry Using Conventional and Serial X-Ray Protein Crystallography

**DOI:** 10.3390/cryst12111671

**Published:** 2022-11-19

**Authors:** Nathan Smith, Mark A. Wilson

**Affiliations:** Department of Biochemistry and the Redox Biology Center, University of Nebraska, Lincoln, NE 68588 USA

## Abstract

Proteins that use cysteine residues for catalysis or regulation are widely distributed and intensively studied, with many biomedically important examples. Enzymes where cysteine is a catalytic nucleophile typically generate covalent catalytic intermediates whose structures are important for understanding mechanism and for designing targeted inhibitors. The formation of catalytic intermediates can change enzyme conformational dynamics, sometimes activating protein motions that are important for catalytic turnover. However, these transiently populated intermediate species have been challenging to structurally characterize using traditional crystallographic approaches. This review describes the use and promise of new time-resolved serial crystallographic methods to study cysteine-dependent enzymes, with a focus on the main (M^pro^) and papain-like (PL^pro^) cysteine proteases of SARS-CoV-2 as well as other examples. We review features of cysteine chemistry that are relevant for the design and execution of time-resolved serial crystallography experiments. In addition, we discuss emerging X-ray techniques such as time-resolved sulfur X-ray spectroscopy that may be able to detect changes in sulfur charge state and covalency during catalysis or regulatory modification. In summary, cysteine-dependent enzymes have features that make them especially attractive targets for new time-resolved serial crystallography approaches, which can reveal both changes to enzyme structure and dynamics during catalysis in crystalline samples.

## 1. Why are cysteine residues interesting?

Of the twenty canonical proteinogenic amino acids, cysteine (Cys) is one of the most reactive at physiological conditions. As a consequence of its reactivity, Cys can play multiple important roles in proteins: catalytic residue in enzyme active sites, favored site for post-translational modifications, ligand in metal binding sites, and disulfide formation (Table 1). The solution pK_a_ of the Cys thiol is approximately 8.5, meaning that only about 3% of unperturbed Cys residues are ionized to the more reactive thiolate anion at pH 7. However, the microenvironment of Cys residues in proteins can lead to depression of the thiol pK_a_ to as low as ~3.3 [1], leading to a greatly increased fraction of thiolate anion for those residues at physiological pH. Cys thiolates are more nucleophilic than thiols and are thus typically the reactive species in Cys chemistry. However, the rate of thiolate reaction with electrophiles is maximal when the thiol pK_a_ matches the solution pH owing to the high transition state barrier to covalently modifying a highly stabilized thiolate anion [2]. Because the protein microenvironment exerts a large influence on Cys ionization and reactivity [3–6], the details of protein structure around Cys residues direct the chemistry that can occur at the Cys. In addition to standard X-ray and neutron crystallographic techniques to characterizing protein structure, new serial crystallography experiments offer time-resolved views of enzyme active sites during catalysis [7–13]. These new classes of experiments are particularly well-suited to following the kinds of covalent catalysis that occur in Cys-dependent enzymes, which typically generate defined intermediates amenable to experimental and computational characterization.

## 2. Cysteine residues in enzyme catalysis: SARS-CoV-2 proteases

**Figure 1.**
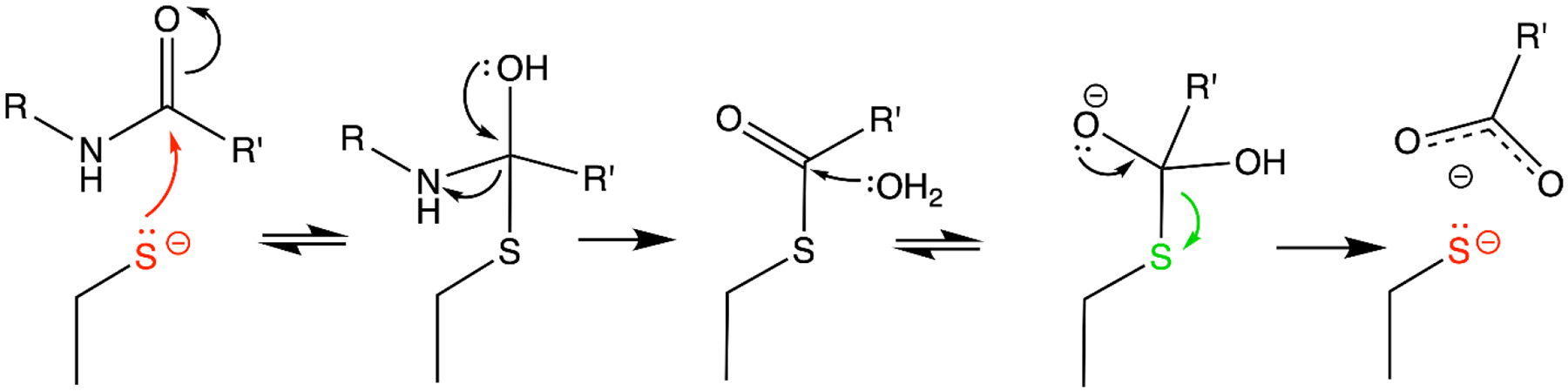
Changes in cysteine nucleophilicity accompanying enzyme catalysis. A generic peptidolytic mechanism is shown, with electron flow indicated with curved arrows and the direction of reaction indicated with straight arrows. In the initial steps of the reaction, the Cys thiolate (red) needs to be nucleophilic enough to form a bond (and the first intermediate) with the substrate. In later steps, the Cys needs to be electrophilic enough to serve as a leaving group (green) and release product. The relative change in degree of Cys nucleophilicity is a combination of the chemical properties of the substrate/intermediate/product as well as structural and electrostatic changes in the Cys microenvironment during catalysis that facilitate enzyme turnover.

Many enzymes feature Cys residues in their active sites, typically serving as a catalytic nucleophile. Cys-dependent enzymes perform a wide variety of essential functions within the cell and include proteases, antioxidant enzymes, kinases, phosphatases, transferases, hydrolases, lyases, isomerases, and ligases. A comprehensive review of Cys-dependent enzymes would exceed the scope of this topical minireview, however a general theme is that they feature a bipartite reaction mechanism. The reaction is initiated by nucleophilic Cys attack to form a covalent intermediate followed by resolution of the catalytic intermediate and release of product via hydrolysis or transfer to another nucleophile. Importantly, there are conflicting chemical demands on catalytic Cys residues throughout the reaction cycle. In the early steps, the Cys should be a good nucleophile and thus ionized but not highly stabilized as a thiolate. Once the catalytic intermediate has formed, the Cys must now be a leaving group with a lower propensity for covalent bond formation (Fig 1). Therefore, efficient Cys-dependent enzymes must change features of the active site microenvironment during catalysis to favor sequentially better and poorer nucleophilic character at the Cys residue as the reaction proceeds. Importantly, nucleophilicity is not an absolute quantity and thus both the detailed active site environment and the electronic configuration of the substrate, intermediate(s), and product influence the behavior of the Cys sulfur atom throughout catalysis. Because the relevant catalytic intermediates are transient, new time-resolved X-ray crystallographic approaches are particularly well-suited to determining how Cys-dependent enzymes accomplish this complex chemical feat and will be discussed in this review.

### 2.1. Chymotrypsin-like Main protease (3CL^pro^ or M^pro^)

Cys chemistry plays a critical role in the lifecycle of severe acute respiratory syndrome coronavirus 2 (SARS-CoV-2), the causative agent of COVID-19 and the focus of this special issue. Table 2 presents the total Cys counts and percentages for each protein in the SARS-CoV-2 proteome, with annotations for functionally important Cys where known. Several proteins from SARS-CoV-2 have markedly higher Cys abundance compared to the overall frequency of 2.2% Cys in all mammalian proteins [14], [15]. One of these Cys-rich proteins is the main or chymotrypsin-like protease (M^pro^ or 3CL^pro^), which has been the subject of a large amount of structural study [16–25]. M^pro^ cleaves several polypro-proteins into their mature form, including eleven cleavage sites in two large polyproteins; replicase 1a and 1ab that are required for viral replication [19] [16]. M^pro^ is essential for viral maturation and is thus an attractive pharmacological target. M^pro^ is targeted by nirmatrelvir, which in combination with ritonavir is marketed under the tradename paxlovid has received FDA emergency use approval for treatment of COVID-19. However, many other drug candidates targeting M^pro^ are being developed [26–28], reflecting the consensus that this Cys protease is one of the most promising targets for the treatment of COVID-19.

**Figure 2.**
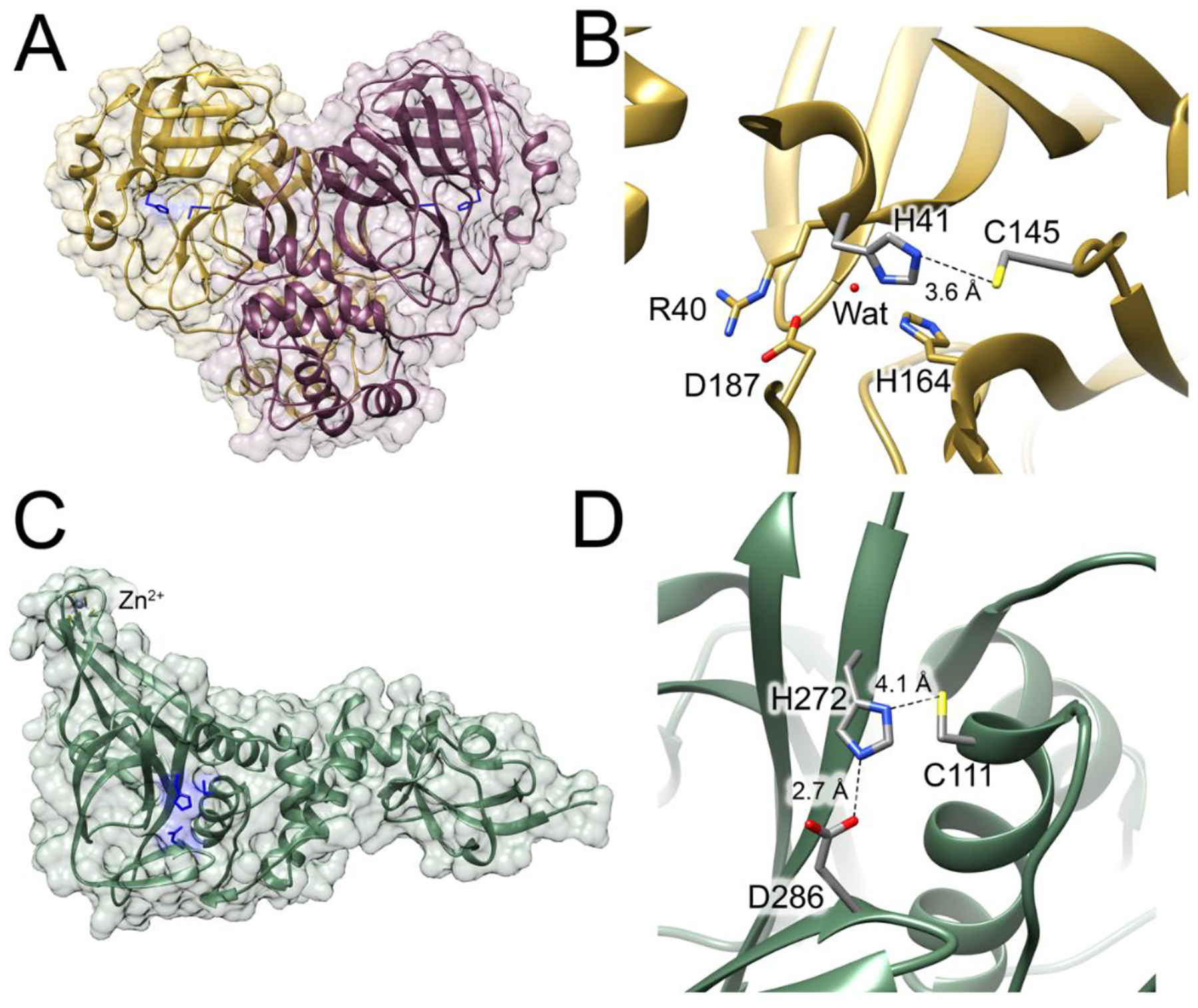
Cysteine-containing active sites in SARS-CoV-2 M^pro^ and PL^pro^. (**A**) M^pro^ (PDB code 6Y2E [21]) shown as a ribbon diagram with active site catalytic dyad in blue. (**B**) A close-up view of the M^pro^ active site with key catalytic dyad residues in grey and other residues identified as important in gold. Key hydrogen bonds are shown as dotted lines with distances given in Ångstroms. (**C**) PL^pro^ (PDB code 6WZU [47]) shown as a ribbon diagram with active site residues in blue and a structural Zn^2+^ site labeled. (**D**) A close-up view of the PL^pro^ active site with the catalytic triad residues in grey. Key hydrogen bonds are shown as dotted lines with distances given in Ångstroms.

The M^pro^ literature has been the subject of many reviews [28–31] and thus we focus on only a few key issues with respect to its Cys active site. The X-ray crystal structure of M^pro^ has been determined at resolutions ranging from 1.2–3.0 Å and revealed that its active site is centered on Cys145, which forms a catalytic dyad with His41 [21,22] (Fig. 2A,B). Catalytic dyads are found in many other Cys proteases [32] and reflect the relative ease with which Cys can be deprotonated compared to higher pK_a_ hydroxylated active site nucleophiles such as serine, where a Ser-His-Asp/Glu catalytic triad is typically used. However, the ionization state of the M^pro^ active site has been debated, with some studies of the related main protease from SARS-CoV-1 (the causative agent of SARS) suggesting that the catalytic dyad may be neutral in the resting enzyme and that Cys145 ionizes to the more nucleophilic thiolate only upon substrate binding [33]. Other studies of SARS-CoV-1 M^pro^ proposed that the dyad is ionized to an imidazolium-thiolate pair (His^+^-Cys^−^) in resting M^pro^ [34]. Neutron crystallography of SARS-CoV-2 M^pro^ settled this issue by directly observing hydrogens (deuterons) on the doubly protonated cationic His41 and no corresponding nuclear density near Cys145 in 2.4 Å resolution nuclear density maps, supporting the existence of an imidazolium-thiolate pair that is poised for nucleophilic attack in the resting enzyme [20]. This is an example of the subtle interplay of hydrogen bonding and electrostatic effects frequently found in Cys-containing active sites which can profoundly influence Cys chemistry in these enzymes [6,18].

The structural biology of M^pro^ illustrates another common theme in X-ray crystallographic studies of catalytic Cys residues in proteins: their tendency towards oxidation. Functionally important Cys residues often have depressed pK_a_ values and thus exist as more reactive thiolates at physiological pH. While this enhances their reactivity towards substrates and regulatory molecules, it also increases the probability of off-pathway chemistry. In M^pro^, a 1.80 Å resolution room temperature X-ray crystal structure showed that the catalytic Cys145 nucleophile can be oxidized to an unusual peroxysulfenate (Cys-S-O-O^−^) at pH 7.0 [19]. No such modification is observed at pH 6.0, where a larger proportion of Cys145 is in the less reactive thiol form [19]. While the physiological significance of this modification is uncertain, Cys oxidation can mimic several active site microenvironment changes that occur during catalysis in these enzymes [35]. Cys modifications are often facilitated by X-ray-induced radiation damage and are typically more pronounced when higher doses are absorbed at synchrotron sources. X-ray crystallographers studying Cys-dependent enzymes should be aware of the enhanced tendency of low pK_a_ Cys residues to be directly modified by oxygen-containing species in the presence of X-rays. This type of modification has been less studied than other types of site-specific radiation damage such as the breaking of disulfide bonds, decarboxylation of aspartate and glutamate residues, loss of the -OH group of tyrosine, oxidation of methionine, [36] and reduction of metal centers [37–40].

### 2.2. Papain-like protease (PL^pro^)

PL^pro^ is another essential SARS-CoV-2 Cys protease because it cleaves three pro-proteins into their mature forms: Nsp1, Nsp2, and Nsp3 [41]. The related PL^pro^ from SARS-CoV-1 (causative agent of SARS) has additional roles related to its canonical proteolytic activity, including deubiquitination and deISGylation [42,43], some of which appear to be shared by the 83% identical PL^pro^ from SARS-CoV-2 [44–46]. Like its namesake, papain, PL^pro^ contains a catalytic triad: Cys111, His272, and Asp286 [47] (Fig. 2B,C), although the acidic residue in the triad is thought to be minor contributor to catalytic activity in papain-like Cys proteases [48] [49]. However, Cys plays another important role in PL^pro^ as a metal ligand, with Cys189, Cys192, Cys224, Cys226 coordinating a Zn^2+^ ion that is essential for activity in both SARS-CoV-1 and SARS-CoV-2 [42,50,51] although it is distant (~40 Å) from the active site. Like M^pro^, PL^pro^ has been the subject of intensive structural study using primarily X-ray crystallography and structures have been determined at resolutions ranging from 1.4–3 Å [47,52–57]. Unlike M^pro^, PL^pro^ has not been studied using neutron crystallography so the ionization state of its catalytic triad is presumed to be the resting thiolate (Cys^−^)-imidazolium (His^+^)-carboxylate (Asp^−^) state found in other papain-like proteases [58,59]. Notably, this model has been challenged in prior computational studies of related enzymes that suggest a thiol (Cys)-imidazloium (His^+^) pair in papain [60] and the related Cys protease cruzain appears to possess a neutral thiol (Cys)-imidazole (His) pair in the resting enzyme that is ionized prior to catalysis in a substrate-assisted mechanism that may be facilitated by a conformational change in the active site [61]. The continued salience of these debates about fundamental aspects of active sites in a thoroughly studied class of Cys protease highlights the sensitivity of Cys ionization to its structural microenvironment, its central importance for enzyme function, and the dangers of generalizing from one example to an entire class of Cys-dependent enzymes.

## 3. New X-ray crystallographic approaches to study Cys chemistry

X-ray crystallography has been the dominant method of macromolecular structure determination for over half a century, although advances in cryo electron microscopy (cryoEM) now provide a powerful new suite of experimental tools for imaging macromolecules [62]. Throughout nearly all its history, X-ray data collection from macromolecular crystals has been performed by measuring multiple diffraction images from a single crystal that is being oscillated or precessed in the X-ray beam. This results in the deposition of a considerable X-ray radiation dose in the sample and the consequent damage can alter the molecule (see discussion of M^pro^ above). In addition, it can be difficult to alter single crystals during X-ray diffraction experiments in ways that are physiologically relevant, for example by introducing substrate into an enzyme crystal. The advent of serial crystallography, whereby a single diffraction image is recorded per crystal and the complete dataset is created by combining images collected from a large number of crystals, has opened up new areas of investigation by allowing enzyme catalysis to be observed in crystalline samples under conditions that were previously infeasible [8,10–12,63,64].

### 3.1. Serial crystallography is an emerging technology for mechanistic enzymology

X-ray Free Electron Lasers (XFELs) produce pulses of coherent X-rays with peak brightness that is nine orders of magnitude greater than synchrotron storage ring radiation and last only 10–100 femtoseconds [7,65]. The field of XFEL macromolecular crystallography has been extensively reviewed [38,65,66] and thus we only touch on a few pertinent points here. Because each pulse of XFEL radiation contains ~10^12^ photons, diffraction data can be collected serially from a large number of microcrystals, each of which is typically destroyed by these high intensity X-ray pulses [67–69]. The resulting composite serial crystallographic dataset has minimal radiation damage, as each crystal was illuminated with X-rays and diffracted before sample destruction set in [70]. Therefore, XFEL serial crystallography dramatically reduces radiation damage compared to traditional crystallographic approaches, which is important for certain Cys-dependent enzymes [10,71] as well as many metalloproteins and other radiation-sensitive samples [36,37,39,66]. Challenges that remain include optimizing the processing of serial crystallography datasets, reducing sample consumption, and the limited amount of XFEL beamtime worldwide. Global XFEL beamtime is constrained by the small number of these facilities (five at present) and their linear geometry, which limits the number of endstations compared to the circular geometry of synchrotron storage rings, which can accommodate many more. However, serial X-ray crystallography has been adapted for synchrotron use and sample consumption has been reduced [13,72–74], positioning the technique to become more widely used for structural study in the years ahead.

### 3.2. Time-resolved serial crystallography of Cys-dependent enzymes

**Figure 3.**
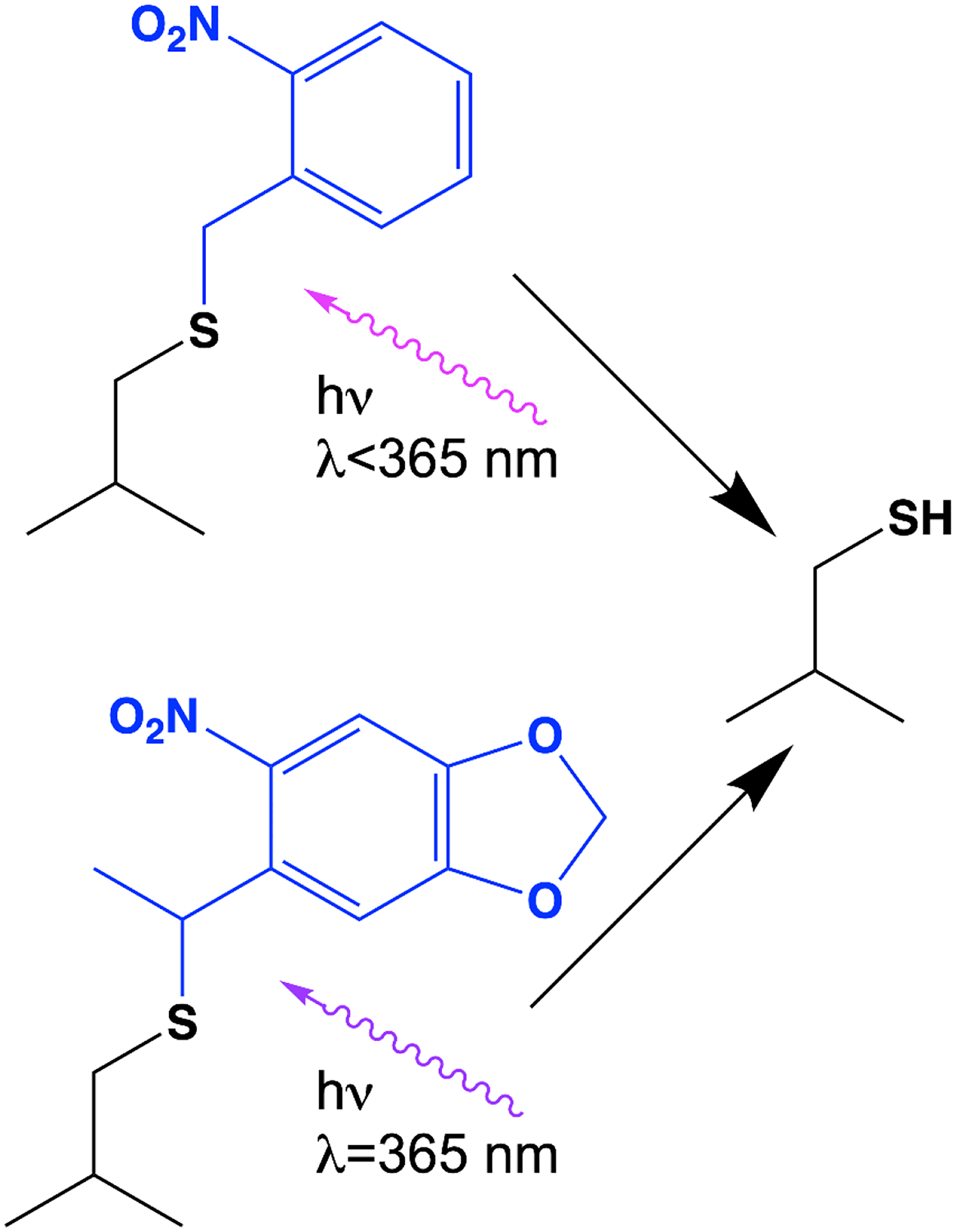
Photocaged Cys residues for activation of Cys-dependent enzymes. Two non-canonical photocaged Cys residues from ref. [84] are shown with wavelengths of the UV light needed to release Cys shown. Note that the lower photocage can be activated at ~365 nm, where protein UV absorption is not expected to interfere.

Microcrystals’ small size and greater tolerance for perturbation [9,10,67,75] means that reactions can be initiated in them more easily and homogeneously than in larger crystals, allowing macromolecular structural changes to be monitored as a function of time by serial crystallography. For enzymes, the most functionally important perturbation is the introduction of substrate that initiates catalysis. There are currently three ways this can be accomplished in serial crystallography: by native photoinitiation, by liberation of a photocaged substrate, or by direct mixing with substrate. Each can be used to initiate catalysis at a defined time across the many molecules in the crystal lattice followed by monitoring the structures of the protein at defined timepoints after catalysis begins. Systems that are sensitive to light are ideal candidates for photoinitiation followed by time-resolved crystallography and include photosystem II [12,67,76], fatty acid photodecarboxylase [11], and photolyase [77] as well as a number of photoproteins that are not enzymes [78]. However, enzymes whose reactions are initiated by light are rare. An alternative is to use a photosensitive compound that will be cleaved by light and liberate the substrate or inhibitor [79,80]. This has been used for fluoroacetate dehalogenase [13], and P450nor [81]. For Cys-dependent enzymes, a photoremovable ruthenium 2,2′-bipyridine (Ru^II^(bpy)_2_) moiety has been designed to release a nitrile-based Cys protease inhibitor upon illumination [82]. Although this compound was not deployed in a serial crystallography experiment and may be too large to be accommodated in certain crystal lattices, it provides a useful conceptual starting point for developing similar photoremovable groups for studying time-dependent structural responses to catalysis or inhibition of other Cys proteases, including M^pro^ and PL^pro^. In addition, the converse approach to photoinitiation of catalysis may be taken, whereby the protein is modified with a photoremovable group on a key amino acid (Fig. 3). Genetically encoded photoremovable Cys residues have been developed [83,84] and sophisticated systems for introducing other photoremovable or photoactivatable amino acid residues have been reported [85]. In this approach, light removes a caging group from a non-canonical amino acid, thereby generating the active enzyme in the crystal that is already suffused with substrate and thereby initiates catalysis. Although the small size of microcrystals facilitates illumination of all the molecules in the lattice, a high quantum yield for the photoreaction is often needed to generate the desired species at sufficient concentration for interpretation of time-resolved crystallography data. Proteases present challenges for time-resolved crystallographic studies of catalysis, as their substrates (peptides and proteins) are typically too large to fit into a crystal lattice. However, it may be possible to accommodate small peptides of a few amino acid residues into some protease crystal lattices and then initiate catalysis with either a photoactivatable substrate or enzyme.

Mixing substrate directly with microcrystals and then injecting this mixture into the X-ray beam is the approach with the broadest application for initiating enzyme catalysis in serial crystallography [86]. Despite the wide applicability of mix-and-inject serial crystallography (MISC) experiments, they are still subject to the fundamental constraints that accompany all time-resolved crystallography experiments: the reaction must occur in the crystal, not damage the crystal, and proceed at rates that can be measured using current technology. Because a substrate or ligand is being soaked into the crystal, there is an upper limit on substrate size imposed by the crystal lattice. This limit is dependent on the solvent content of the crystal being studied and dimensions of its lattice voids, but is in the approximate range of 500–1000 Da. As reviewed by Schmidt [86], it takes approximately 1 ms for a ~200 Da substrate to uniformly permeate a 3×4×5 μm^3^ crystal and this time changes by the square of the change in crystal linear dimension, providing a useful approximate guide for estimating the diffusion-limited timescales for MISC. Since microcrystals are frequently in the ~10 μm range, diffusion homogenizes substrate concentration in the crystal in about 15 ms, well within the kinetic regime of many reactions *in crystallo* [86]. However, enzymes with turnover numbers that exceed ~50 sec^−1^ *in crystallo* are challenging to characterize with standard MISC approaches because of these diffusion-limited rates and thus make good candidates for photoinitiation with caged substrates or enzymes.

**Figure 4.**
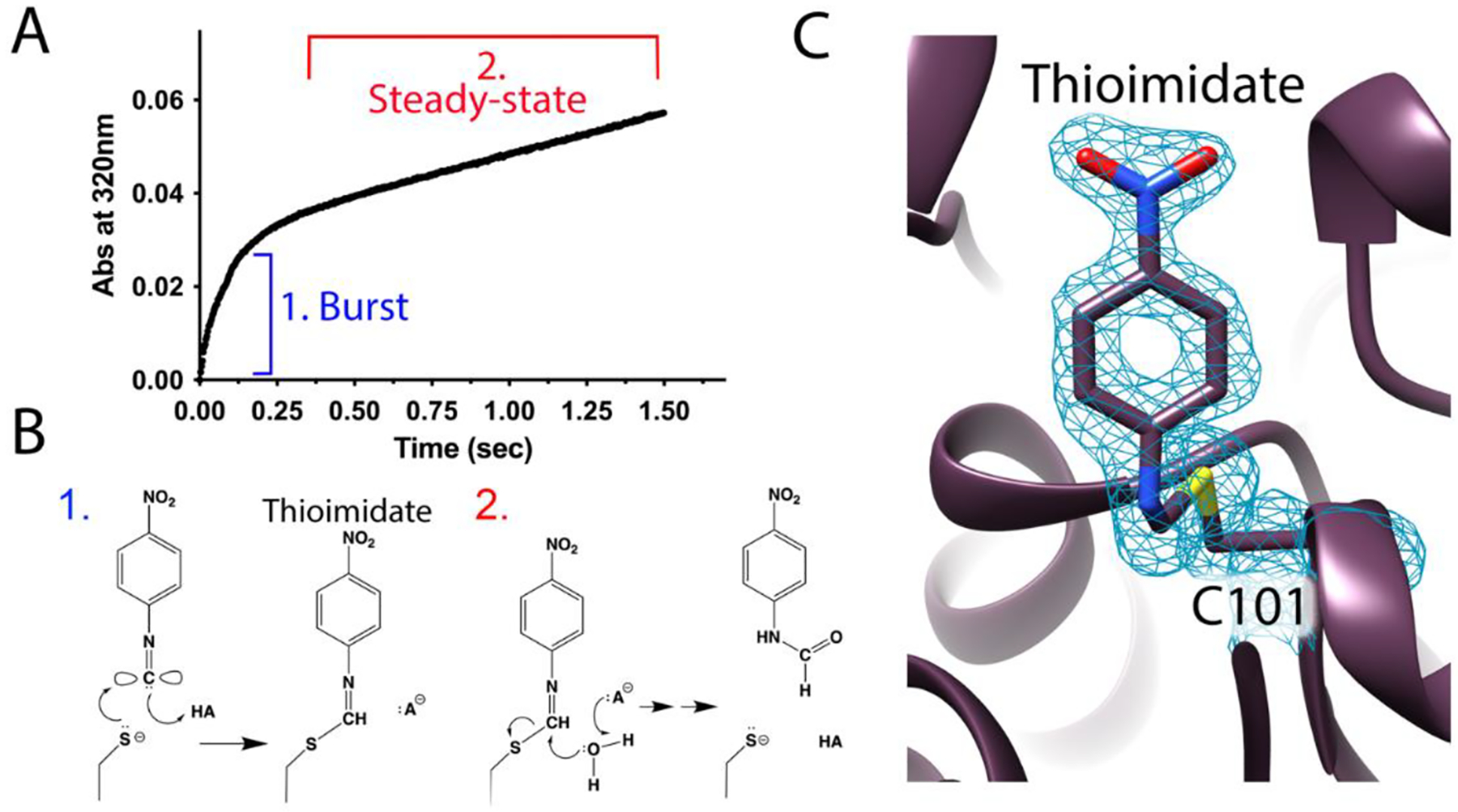
Burst kinetics facilitates intermediate observation in time-resolved crystallography. (**A**) An example of burst kinetics observed in pre-steady state enzyme kinetics of *Pseudomonas fluorescens* isocyanide hydratase (ICH), with the kinetically distinct portions labeled and colored. (**B**) An illustration of the dominant reaction step being monitored in the burst phase (1, blue) and at steady state (2, red). Note that both the thioimidate intermediate and the N-formamide product absorb light at 320 nm, so both species are contributing to the absorbance. In 2, it is not known with certainty if thioimidiate hydrolysis or product release is rate-limiting, although time-resolved crystallography suggests that it is thioimidiate hydrolysis. (**C**) The thioimidate intermediate is clearly resolved in 1.55 Å resolution 2mF_o_-DF_c_ electron density contoured at 0.9σ observed 15 seconds after introduction of para-nitrophenyl isocyanide substrate in a mix-and-inject serial crystallography experiment [10]. The accumulation of the thioimidate during the burst phase makes observation by time-resolved X-ray crystallography more likely.

Injectors that permit MISC in liquids or related approaches that use mixing on solid crystal supports have been deployed at both XFEL and synchrotron X-ray sources [8,73,87–91]. At the time of writing, no MISC experiments on SARS-CoV-2 targets have been published, although there are lessons that can be learned from considering MISC on another Cys-dependent enzyme, isocyanide hydratase (ICH) [10]. ICH possesses several features that are shared among most Cys-dependent enzymes and which make them an attractive class of targets for time-resolved serial crystallography. Most Cys-dependent enzymes use covalent catalytic strategies, and therefore it is likely that intermediates can be observed in many of them using time-resolved serial crystallography. For ICH, the proposed covalent intermediate was a Cys thioimidate [71,92]. In the case of Cys proteases like M^pro^ and PL^pro^, the acyl-enzyme intermediate formed after nucleophilic attack of the active site Cys residue at the substrate scissile bond would be the most likely species to be observed. Second, ICH exhibits burst kinetics, whereby the initial nucleophilic attack by the active site thiolate is faster than later steps, which are rate-limiting (Fig. 4A,B). The burst is only experimentally observed if signal is generated from the species (intermediate or product) that is formed by the faster catalytic steps. Not all Cys-dependent enzymes will exhibit burst kinetics under all conditions, but their common formation of covalent intermediates means that most of these enzymes could have kinetic bursts under appropriate reaction conditions and measurement protocols. Enzymes operating under burst kinetics regimes are expected to accumulate intermediates at higher occupancies than those that do not operate in the burst regime, facilitating interpretation of the time-dependent electron density maps generated by these experiments [10,93]. In ICH, this burst led to the formation of a highly occupied thioimidate catalytic intermediate in well-defined electron density at 1.55 Å resolution 15 seconds after mixing with substrate (Fig. 4C). Third, the requirement for Cys thiolate formation for catalytic activity in most Cys-dependent enzymes means that reaction rates can often be altered by modest adjustments of solution pH. However, it should be borne in mind that while pH is a useful control parameter that can adjust crystalline enzymes reaction rates to suit an experiment [94–96], crystals are often sensitive to pH and thus the limits of a crystal’s tolerance for pH perturbation must be established for each system. Fourth, reactive Cys residues are frequently subject to off-pathway modifications, especially oxidation, and these events can be caused by X-ray radiation. While sometimes informative [19,35], such modifications can often interfere with interpretation of electron density features around the catalytically important Cys residue. Serial crystallography dramatically reduces the effective absorbed radiation dose in a dataset [70,97] and thus can circumvent site-specific X-ray damage to sensitive Cys residues. In summary, many Cys-dependent enzymes possess mechanistic and kinetic features that make them good candidates for successful time-resolved crystallography experiments and there are sound a priori technical reasons to prioritize their study using these techniques.

### 3.3. Sulfur X-ray spectroscopy to monitor electronic state of Cys

The development of time-resolved X-ray spectroscopy at synchrotrons and XFELs has provided the ability to monitor sulfur electronic states in real time. Sulfur covalently bonds with many atoms in diverse ways, reflected in the range of oxidation states that it can adopt. Consequently, X-ray spectroscopy can monitor changes to the electronic state of sulfur atom as its charge or degree of covalency change during enzyme catalysis. In some cases, the changes in sulfur’s electronic state can be reliably inferred from inspection of electron density maps that show formation of new bonds in covalent intermediates. However, other changes to sulfur’s valence shell electronic configuration are difficult or impossible to determine via X-ray crystallography, such as the ionization of a thiol to a thiolate, the formation of a thiyl radical, or changes in formal charge on sulfur in metal complexes such as iron-sulfur clusters. While UV-visible spectroscopy can report on certain sulfur transitions [98–100], following changes in sulfur charge/valency states in macromolecules using UV-visible spectroscopy in the 240 nm region is complicated by protein and nucleic acid absorption interference. For these reasons, using X-ray absorption spectroscopy (XAS) and X-ray emission spectroscopy (XES) to monitor changes in Cys valency and charge is appealing and can be coupled to other structural experiments [101,102]. Demonstrating its potential for broad biological application, sulfur X-ray spectroscopy has been used to characterize samples ranging from small molecules to tissue sections [102,103]. Because K-edge X-ray excitations of sulfur raise its core electrons to the valence shell, XAS/XES can report on subtle differences in the valence shell electron configuration of sulfur in different compounds. Femtosecond XFEL sources and energy-dispersive von Hamos spectrometers could, in principle, measure enough tender X-ray photons per pulse to make “single-shot” sulfur XAS/XES possible [104], as they have done for metals [102]. Time-resolved X-ray sulfur spectrometry has been used to study molecular dynamics following photoexcitation of 2-thiopyridone [105], which lays the groundwork for potential time-resolved application to larger molecules. A major challenge in applying this technique to proteins will be obtaining the necessary signal-to-noise ratio in the XAS/XES spectra to discern changes in a single active site sulfur atom that is changing during the reaction amidst a background of multiple “bystander” sulfur atoms in Cys and Met residues that are not involved in catalysis. Although there are significant experimental hurdles to be overcome in order to apply single-shot sulfur XAS/XES to proteins such as M^pro^ and PL^pro^, the value of being able to monitor the electronic states of Cys sulfur residues in real time throughout enzyme catalysis will undoubtedly motivate additional work in this direction. Sulfur XAS/XES could be especially powerful if performed on microcrystals that could be used in parallel time-resolved serial X-ray crystallography experiments.

## 4. Targeting Cys residues for covalent modification

A central motivation for studying Cys-dependent enzymes is understanding how to inhibit them. As discussed above (Section 2.1), covalent targeting of the active site Cys in M^pro^ is a current front-line strategy for COVID-19 treatment and the development of these inhibitors was guided by structural considerations [28,31]. Covalent inhibitors offer several advantages over their non-covalent kin, including extremely high apparent affinity for their targets and long-lasting inhibition [27,106,107]. Cys is an attractive target for covalent inhibitors because it is nucleophilic and comparatively rare, diminishing the probability of off-target effects that might arise if more inherently reactive compounds were used to target less nucleophilic, more common residues [108,109]. The irreversible modification of target Cys residues has been used to modulate the activity of several enzymes, such as kinases, ubiquitin conjugation enzymes, phosphatases, ATPases, proteases and others [109–115]. Given the prominence of Cys proteases in SARS-CoV-2 lifecycle and the abundance of Cys residues in the viral proteome, the promise of Cys-directed covalent inhibitors for SARS-CoV-2 treatment is clear. Time-resolved crystallography can be used to directly observe the modification of target Cys residues and map the resulting changes in protein structure. Observing the response of a protein’s conformational ensemble to inhibitor binding is conceptually similar to applications of time-resolved crystallography to enzyme catalysis and may help address unresolved questions about the applicability of conformational selection and induced fit models to ligand binding in various proteins.

Not all functionally important Cys residues are in enzyme active sites. Noncatalytic Cys residues can targeted by compounds that result in conformational changes that inhibit the target protein and are specific for their targets because the modified Cys is often more poorly conserved than active site Cys residues [116–120]. Allosteric Cys-directed inhibitors can sometimes recapitulate the action of natural allosteric modifiers, including glutathione, nitric oxide, reactive oxygen species, and others [109]. A potential application of time-resolved crystallography is using the conformational changes that occur during catalysis to identify transiently exposed allosteric sites that might be targeted. The highly Cys-rich SARS-CoV-2 proteome suggests that there may be a large number of candidate non-catalytic Cys residues that could be so targeted.

## 5. Summary

This brief, targeted review has aimed to draw the reader’s attention to areas of Cys-dependent enzymology where new crystallographic approaches might be fruitfully employed. This review is neither comprehensive of the technology or the potential systems and has (loosely) followed the theme of this special issue with a focus on proteins important for SARS-CoV-2. Our central messages are: 1) catalysis in Cys-dependent enzymes involves a delicate interplay of microenvironmental interactions that stabilize thiolates and reaction intermediates, which sometimes require active site residues to sample different conformations during catalysis, 2) that Cys-dependent enzymes make especially attractive targets for time-resolved serial crystallography because their catalytic mechanisms and modes of inhibition often involve formation of covalent intermediates that are easily observed in electron density maps and, 3) that serial crystallography is a powerful technique to study structural and dynamic events in enzyme catalysis that will likely find broader application in the near future as these experiments become easier to conduct and interpret.

## Figures and Tables

**Table 1.** Examples of cysteine states in proteins.

Cys State	Description
R-SH	Sulfhydryl (unmodified; pK_a_~8.5)
R-SNO	S-nitrosation
R-S-OH	Sulfenic acid/sulfenate (pK_a_~6)
R-S-OOH	Sulfinic acid/sulfinate (pK_a_~2)
R-S-OOOH	Sulfonic acid/sulfonate (pK_a_~−2)
R-S-S-G	Glutathionylation
R-S-S-R’	Disulfide
R-S-Ub	Ubiquitination
R-S-M	Metal binding/coordination
R-S-C-R’	Alkylation
R-S-CO-CH3	Acetylation

**Table 2.** Cys content of the SARS-CoV-2 genome.

Protein	Number of Cys residues; %	Cys of known functional importance^1^	Role of functional Cys
nsp1	1; 0.6	NA	NA
nsp2	27; 4.2	NA	NA
nsp3(PL^Pro^)	10; 3.2	C111*, C189^+^, C192^+^, C224^+^, C226^+^	Nucleophile*, Zn^2+^ binding^+^
nsp4	16; 3.2	NA	NA
nsp5 (M^Pro^)	12; 3.9	C145	Nucleophile
nsp6	10; 3.4	NA	NA
nsp7	3; 3.6	NA	NA
nsp8	2; 1.0	NA	NA
nsp9	3; 2.7	NA	NA
nsp10	13; 9.4	C74, C77, C90, C117, C120, C128, C130	Zn^2+^ binding
nsp11	1; 7.7	NA	NA
nsp12	29; 3.1	C301, C306, C310, C487, C645, C646	Zn^2+^ binding
nsp13	26; 4.3	NA	NA
nsp14	23; 4.4	NA	NA
nsp15	5; 1.4	NA	NA
nsp16	5; 1.7	C115	adenosine stabilization
E Protein	3; 4.0	NA	NA
M protein	4; 1.8	NA	NA
S protein	40; 3.1	NA	NA
N protein	0; 0.0	NA	NA
orf3a	7; 2.5	NA	NA
orf3b	0; 0.0	NA	NA
orf6	0; 0.0	NA	NA
orf7a	6; 5.0	NA	NA
orf7b	2; 4.7	NA	NA
orf8b	7; 5.8	NA	NA
orf9b	0; 0.0	NA	NA
orf9c	5; 7.4	NA	NA
orf10	1; 2.6	NA	NA

Numbering is provided for the mature (i.e. processed) peptide. “NA” is not applicable or not yet known.
